# In Vivo and In Vitro Characterization of Close Analogs of Compound KA-11, a New Antiseizure Drug Candidate

**DOI:** 10.3390/ijms24098302

**Published:** 2023-05-05

**Authors:** Marta Andres-Mach, Mirosław Zagaja, Joanna Szala-Rycaj, Aleksandra Szewczyk, Michał Abram, Marcin Jakubiec, Katarzyna Ciepiela, Katarzyna Socała, Piotr Wlaź, Gniewomir Latacz, Nadia Khan, Krzysztof Kaminski

**Affiliations:** 1 Department of Experimental Pharmacology, Institute of Rural Health, Jaczewskiego 2, 20-950 Lublin, Poland; 2 Department of Medicinal Chemistry, Faculty of Pharmacy, Jagiellonian University Medical College, Medyczna 9, 30-688 Krakow, Poland; 3 Department of Animal Physiology and Pharmacology, Institute of Biological Sciences, Faculty of Biology and Biotechnology, Maria Curie-Skłodowska University, Akademicka 19, 20-033 Lublin, Poland; 4 Department of Technology and Biotechnology of Drugs, Jagiellonian University Medical College, Medyczna 9 St., 30-688 Krakow, Poland

**Keywords:** antiseizure drugs, drug development, acute seizure models, physicochemical descriptors, leading compound optimization

## Abstract

Epilepsy is a neurological disorder involving a number of disease syndromes with a complex etiology. A properly matched antiseizure drug (ASD) gives remission in up to 70% of patients. Nevertheless, there is still a group of about 30% of patients suffering from drug-resistant epilepsy. Consequently, the development of new more effective and/or safer ASDs is still an unmet clinical need. Thus, our current studies were focused on the structural optimization/modifications of one of the leading compounds, **KA-11**, aiming at the improvement of its antiseizure activity. As a result, we designed and synthesized two close analogs with highly pronounced drug-like physicochemical properties according to in silico predictions, namely **KA-228** and **KA-232**, which were subsequently tested in a panel of animal seizure models, i.e., MES, 6 Hz (32 mA), *sc*PTZ and *iv*PTZ. Among these compounds, **KA-232**, which was designed as a water-soluble salt, was distinctly more effective than **KA-228** and assured similar antiseizure protection as its chemical prototype **KA-11**. With the aim of a more detailed characterization of both new molecules, in vitro binding tests were performed to evaluate the potential mechanisms of action. Furthermore, **KA-232** was also evaluated in several ADME-Tox studies, and the results obtained strongly supported its drug-like potential. The proposed chemical modification of **KA-11** enabled the identification of new pharmacologically active chemotypes, particularly water-soluble **KA-232**, which, despite the lack of better efficacy than the leading compound, may be used as a chemical prototype for the development of new ASDs, as well as substances potentially active in other neurological or neurodegenerative conditions.

## 1. Introduction

The problem of drug resistance affects approximately one-third of patients suffering from epilepsy [1,2,3,4]. Because of this, many multidisciplinary studies are currently focused on the development of new antiseizure drug (ASD) candidates (based on the existing ASDs), characterized by stronger antiseizure properties, better absorption/bioavailability, minimal/zero side effects and a multimodal mechanism of action. The introduction of such modifications to the chemical structure of currently available medications may contribute to the development of new, improved ASDs [5,6,7]. Over the past several years, we have been conducting intensive research on a new hybrid compound, a 2-(2,5-dioxopyrrolidin-1-yl)propanamide derivative, compound **KA-11** (also named C-11), which, as a hybrid molecule, combines structural fragments of three ASDs: ethosuximide, levetiracetam and lacosamide. The results of our previous in vitro studies and animal models of epilepsy clearly showed that the hybrid **KA-11** has strong antiseizure and neuroprotective properties in acute mouse seizure models: maximal electroshock seizures (MES), subcutaneous pentylenetetrazole (*sc*PTZ) seizures, pilocarpine (PILO) seizures and the 6 Hz seizure model [6,8]. Moreover, we have shown that **KA-11** suppresses seizures after repeated administration of PTZ in the mouse kindling model of epilepsy [9]. Subsequent studies using this compound have shown that chronic administration of **KA-11** does not negatively affect the proliferation of neural stem cells in mice [10]. Furthermore, **KA-11** significantly stimulated the proliferation migration and differentiation of newborn cells into neurons and astrocytes, as well as protected the cognitive functions in the mouse PILO-induced status epilepticus (PILO-induced SE) [11]. Finally, our last studies using **KA-11** in combination with several selected ASDs in the mouse model of an MES test indicated a significant pharmacodynamic impact of **KA-11** on the antiseizure action of two ASDs, lacosamide and valproate, with no adverse effects among the tested drugs [8]. Moreover, in silico studies indicated that **KA-11** meets the drug-likeness criteria resulting from Lipinski’s [12] and Veber’s [13] rules, as well as the prediction of gastrointestinal absorption and brain penetration (according to Instant JChem by the ChemAxon software version 21.4.0).

**Figure 1:**
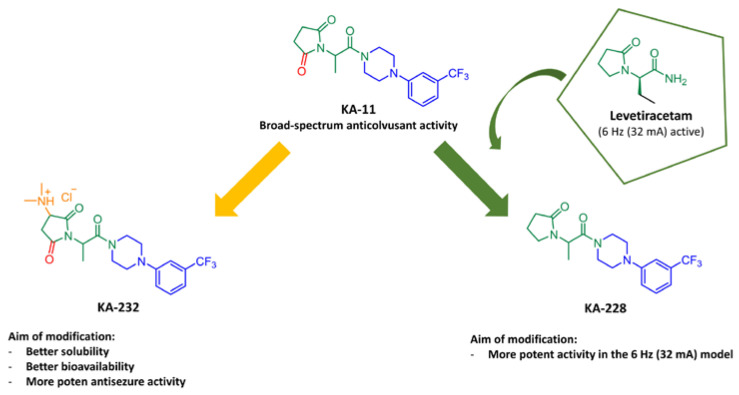
Design strategy and structures of new analogs of **KA-11**–**KA-232** and **KA-228**.

Favorable in vivo, in vitro and in silico results obtained for **KA-11** so far prompted us to design close analogs modified in the pyrrolidine-2,5-dione (succinimide) ring, namely **KA-228** and **KA-232** (Figure 1). It should be emphasized that this is the first attempt at structural combinations focused on succinimide in the series of compounds represented by the leading compound **KA-11**.

The rationale for the proposed structural modifications assumes (*i*) an improvement in efficacy in all seizure models by the preparation of water-soluble hydrochloride (**KA-232**) and, as a result, a hypothetically better pharmacokinetic profile (i.e., higher concentration in the brain) and (*ii*) an exchange of the pyrrolidine-2,5-dione ring to pyrrolidine-2-one moiety with the aim of increasing protection in the 6 Hz (32 mA) seizure model. Notably, the pyrrolidine-2-one core ring is present in the structure of levetiracetam which proved to be especially effective in 6 Hz (32 mA) seizures. Furthermore, the exchange of the succinimide ring to pyrrolidine-2-on lowers the planarity of this fragment which in turn may contribute to better water solubility and an improved pharmacokinetic profile. Obtaining favorable results from the conducted studies, namely the similar or improved antiseizure activity for **KA-228** or **KA-232** vs. parent compound **KA-11**, may contribute substantially to the development of new drug candidates effective in various types of epileptic seizures or the identification of new chemical prototype(s) for further and extensive chemical and pharmacological studies, namely a series of pyrrolidine-2-one (**KA-228** analogs) or water-soluble salts (**KA-232** congeners).

## 2. Results and Discussion

### 2.1. Synthesis of Target Compounds ***KA-232*** and ***KA-228***

**Scheme 1:**
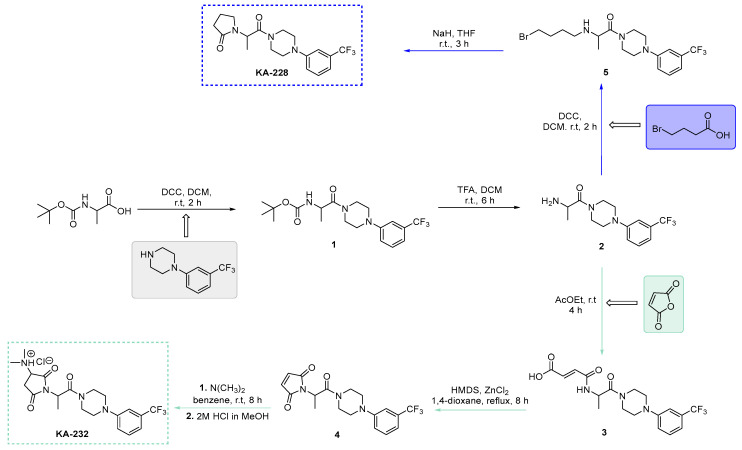
Synthesis of target compounds **KA-232** and **KA-228**.

The water-soluble salt **KA-232** and pyrrolidin-2-one analog **KA-228** were prepared according to the method depicted in Scheme 1. In the first step, the *N*-(tert-butoxycarbonyl)-DL-alanine was coupled in the presence of DCC (*N*,*N*′-dicyclohexylcarbodiimide) with 1-(3-(trifluoromethyl)phenyl)piperazine to give Boc-protected alanine derivative 1. Removal of the Boc protecting group in the presence of trifluoroacetic acid (TFA) yielded amine 2. The first and second steps were common for both **KA-232** and **KA-228**. In the case of salt derivative **KA-232**, amine 2 was converted to amido-acid derivative 3 in the condensation reaction with maleic anhydride. The monounsaturated pyrrolidine-2,5-dione derivative 4 was obtained by applying the hexamethyldisilazane (HMDS)-promoted cyclization reaction of 3. The final compound **KA-232** was obtained in the addition reaction of dimethylamine with intermediate 4. This reaction was performed in benzene at room temperature. Finally, **KA-232** was converted into water-soluble hydrochloride by dissolving it in a 2M methanolic hydrochloric acid solution. The resulting product was obtained in the form of a white solid substance, which was isolated by concentrating the organic solvent under reduced pressure and washing it with diethyl ether. In the case of the pyrrolidin-2-one derivative **KA-228**, the primary amine 2 was coupled with 4-bromobutanoic acid in the presence of DCC to yield derivative 5. Next, compound **5** was cyclized in the presence of base sodium hydride, to give the desired product **KA-228**.

### 2.2. In Silico Determination of Selected Physicochemical Descriptors

The physicochemical properties of **KA-232** and **KA-228** were determined based on Lipinski’s and Veber’s rules using the online tool SwissAdme website [14] (Table 1).

**Figure 2:**
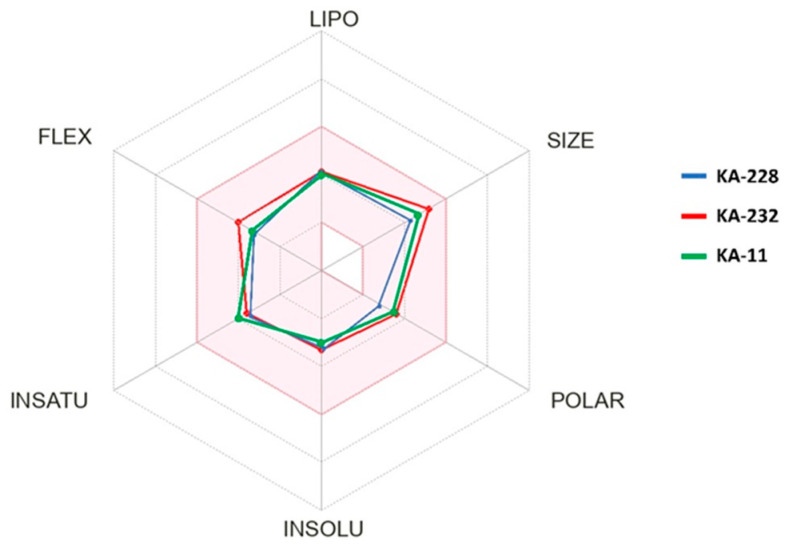
The bioavailability radar of compounds **KA-232**, **KA-228** and parent **KA-11** (LIPO, lipophilicity; POLAR, polarity; INSOLU, solubility; FLEX, flexibility; INSATU, saturation of the molecule). Pink area means range of optimal physicochemical parameters.

Lipinski’s and Veber’s rules are utilized to assess the drug-like characteristics of a chemical compound, which help to determine whether it possesses physicochemical properties favorable for oral absorption in humans. The criteria of Lipinski’s rules are MW ≤ 500 Da, log P ≤ 5, HBD ≤ 5 and HBA ≤ 10, and those of Veber’s rules are NBR ≤ 10 and TPSA ≤ 140 Ǻ^2^ [12,13]. Consequently, according to Table 1, both compounds meet the requirements of both the aforementioned rules. The SwissAdme website also offers radar charts that take into account six physicochemical properties (lipophilicity, size, polarity, solubility, flexibility and saturation) to depict the relationship between the chemical structure of a molecule and its oral bioavailability. These radar charts display color-coded dots that represent the physicochemical properties of **KA-232**, **KA-228** and their parent compound **KA-11**. The acceptable range of physicochemical parameters, as determined by Lipinski’s and Veber’s rules, is indicated by the pink area on the charts (Figure 2). Based on this analysis, it can be concluded that **KA-232** and **KA-228** fulfill the drug-likeness criteria set forth by Lipinski’s and Veber’s rules.

Additionally, **KA-232** and **KA-228**, like their parent compound **KA-11**, meet the requirements of central nervous system multiparameter optimization (CNS MPO) using Instant JChem by the ChemAxon software version 21.4.0. The CNS MPO score is a well-established algorithm that evaluates six key physicochemical properties: ClogP, calculated distribution coefficient at pH 7.4 (ClogD), TPSA, HBD, MW and most basic center (pKa). Each property has a value between 0 and 1, thus giving the maximum collective score of 6. A higher CNS MPO score is preferred, and a score ≥ 4.0 is generally used as a cut-off point for selecting compounds for hit finding in CNS drug discovery programs [15]. It is important to note that **KA-232** and **KA-228** scores were >5.5.

**Figure 3:**
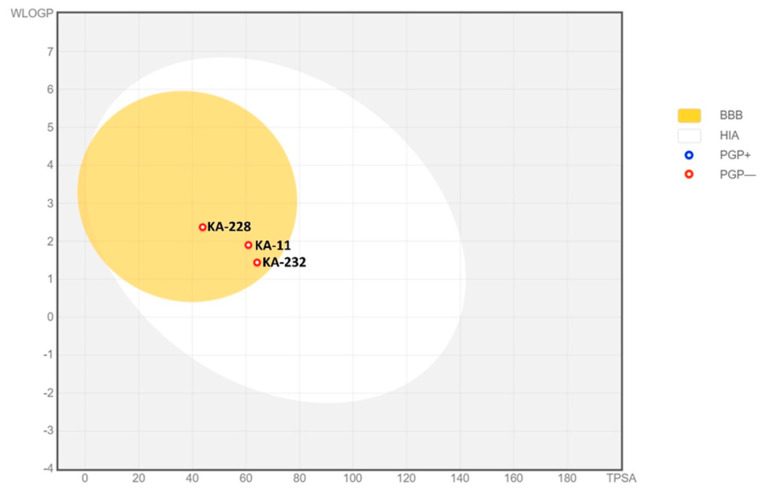
The BOILED-Egg predictive model for **KA-232**, **KA-228** and parent **KA-11** (marked as red dots). The white region represents the range of physicochemical properties favorable for absorption from GI tract (see human intestinal absorption HIA in legend), and the yellow region (see BBB in legend) is the range of physicochemical properties’ favorable penetration through BBB. PGP+, potential substrate for P-glycoprotein (PGP) efflux; PGP−, low risk of interaction with PGP.

In addition to low efficacy or high toxicity, the low bioavailability from the gastrointestinal (GI) tract or weak penetration through the blood–brain barrier (BBB) is one of the reasons for many failures in the development of CNS-active drugs. Therefore, the SwissAdme website offers a tool called ‘BOILED-Egg’ (Figure 3), which predicts how well a substance is absorbed from the GI tract and can penetrate the BBB. According to this model, **KA-232** and **KA-228** have a high likelihood, similar to their parent compound **KA-11**, to be well absorbed from the GI tract and to effectively penetrate through the BBB.

### 2.3. Antiseizure and Safety Evaluations In Vivo

Using animal models of seizures has been recognized as the most important approach for the discovery and development of new ASDs due to their high predictability and good translation of results from rodents to humans. Notably, all clinically relevant ASDs have been identified by applying this paradigm. Interestingly, so far, only padsevonil which is a dual-acting drug (SV2/GABA_A_ ligand), is an example of rationally designed ASDs (development discontinued in Phase 2B) [16]. Consequently, the newly obtained compounds **KA-228** and **KA-232** were tested in a panel of well-established seizure models, namely the MES, 6 Hz (32 mA), *sc*PTZ and intravenous *iv*PTZ tests. Compounds effective in these models may provide protection from different seizures in humans such as tonic–clonic, focal myoclonic and absence seizures. Apart from the antiseizure evaluations, both compounds **KA-228** and **KA-232** were tested in the chimney test and the grip strength test with the aim of assessing their safety profile in mice.

**Figure 4:**
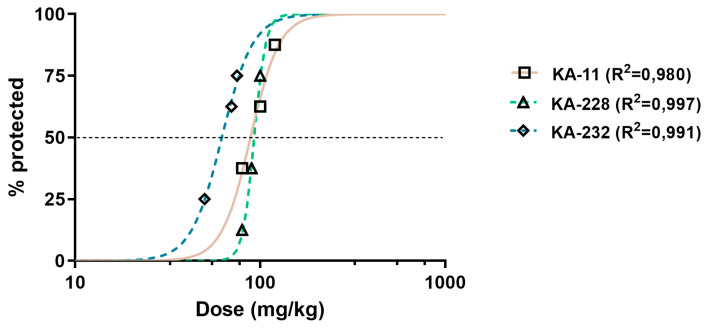
Effects of **KA-11**, **KA-228** and **KA-232** in the MES model in mice. The graph illustrates dose–response functions (sigmoidal curves) for the anticonvulsant activity of the studied compounds. Each data point represents the percentage of mice protected from seizures (*n* = 8 mice/data point). Sigmoidal curves are the result of the least squares fitting of the dose–response function for each compound separately. Points of intersections with the dashed line at 50 represent the approximate ED_50_ values of compounds. R^2^—a coefficient of determination. The results for **KA-11** were used from Kaminski et al. [6] (see compound **11**).

In the MES test, **KA-228** and **KA-232** administered alone protected the animals, in a dose-dependent manner, from tonic–clonic seizures (Figure 4). Their ED_50_ values are presented in Table 2. The ED_50_ value for **KA-232** was significantly lower in comparison to the **KA-11** ED_50_ value obtained previously by Kaminski et al. [6] (61.80 mg/kg vs. 88.40 mg/kg, respectively). In turn, the ED_50_ value for **KA-228** was slightly higher in comparison to **KA-11**; however, the difference was not statistically significant.

A mouse MES seizure model was previously used to evaluate the impact of **KA-11** on the protective efficacy of several selected antiseizure drugs [8]. The obtained results indicated that **KA-11** at an ineffective dose (30 mg/kg, intraperitoneal i.p. injection) significantly enhanced the anticonvulsant action of lacosamide and valproate without affecting their metabolism, and it affected the animal’s behavior when used in combination. Considering the beneficial effect of **KA-11** on the anticonvulsant activity of other ASDs, it might be worth considering similar studies for the **KA-232** analog.

A favorable anticonvulsant effect for the **KA-232** analog in the mouse MES model certainly confirms the importance of the optimization of the already known compounds aiming at the improvement of their efficacy in the MES model which mimics human tonic–clonic seizures, among others [17].

**Figure 5:**
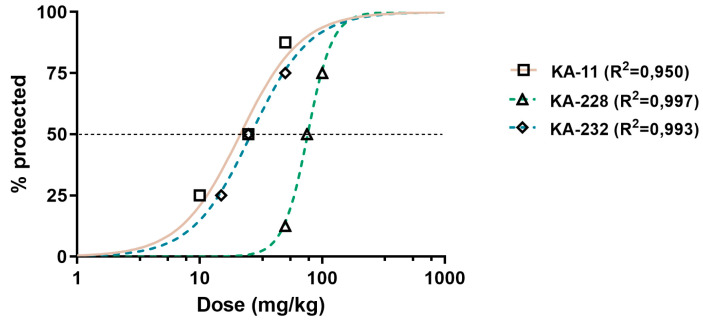
Effects of **KA-11**, **KA-228** and **KA-232** in the 6 Hz (32 mA) test in mice. The graphs illustrate dose–response functions (sigmoidal curves) for the anticonvulsant activity of the studied compounds. Each data point represents the percentage of mice protected from seizures (*n* = 8 mice/data point). Sigmoidal curves are the result of the least squares fitting of dose–response function for each compound separately. Points of intersections with the dashed line at 50 represent the approximate ED_50_ values of compounds. R^2^—a coefficient of determination. The results for **KA-11** were used from Kaminski et al. [6] (see compound **11**).

Further in vivo studies showed that both **KA-228** and **KA-232** administered alone protected the animals, in a dose-dependent manner, from psychomotor seizures in the 6 Hz (32 mA) model of human focal epilepsy [18] (Figure 5 and Table 2). However, the ED_50_ value for **KA-228** was significantly higher in comparison to the previously evaluated value of **KA-11** by Kamiński [6] (61.80 mg/kg vs. 21.00 mg/kg, respectively). On the other hand, the ED_50_ value for **KA-232** was 26.50 mg/kg and was not significantly different from the ED_50_ for **KA-11** in this experimental seizure model.

According to the obtained results, we can observe different anticonvulsant effectiveness of **KA-228** and **KA-232** depending on the type of applied electrical stimulation (MES and 6 Hz). These distinctions may result from a different mechanism of seizure induction in both experimental models (myoclonic seizures in MES and psychomotor seizures in 6 Hz), as well as from a difference in the structure of the tested compounds, which may reflect their pharmacological properties.

Based on the main assumption in the process of modifying **KA-11**, namely that the compound increases antiseizure effectiveness, both analogs did not show an improvement over the **KA-11** compound in the 6 Hz model; nevertheless, it should be stressed that water-soluble analog **KA-232** was almost as effective as **KA-11**.

**Figure 6:**
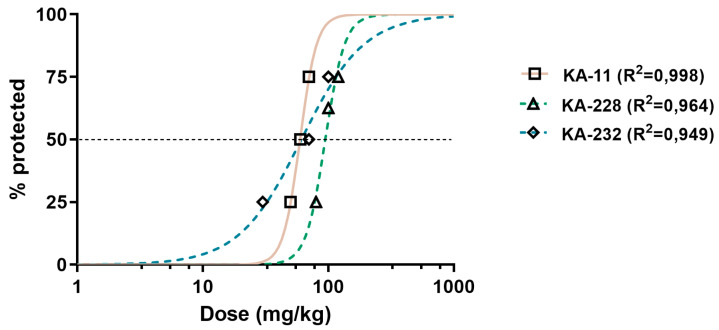
Effects of **KA-11**, **KA-228** and **KA-232** in PTZ-induced clonic seizures in mice. The graphs illustrate dose–response functions (sigmoidal curves) for the anticonvulsant activity of the studied compounds. Each data point represents the percentage of mice protected from seizures (*n* = 8 mice/data point). Sigmoidal curves are the result of the least squares fitting of dose–response function for each compound separately. Points of intersections with the dashed line at 50 represent the approximate ED_50_ values of compounds. R^2^—a coefficient of determination. The results for **KA-11** were used from Kaminski et al. [6] (see compound **11**).

Based on the satisfying protection profile of **KA-228** and especially **KA-232** in the MES seizure model in the next step of pharmacological investigations, both compounds were tested in chemically induced seizures, namely *sc*PTZ and *iv*PTZ models. Consequently, **KA-228** and **KA-232** administered alone protected the animals, in a dose-dependent manner, from clonic seizures in the *sc*PTZ model of absence epilepsy (Figure 6). The ED_50_ value for **KA-228** was significantly higher in comparison to the ED_50_ value of **KA-11**, previously studied by Kamiński et al. [6] (95.40 mg/kg vs. 59.90 mg/kg, respectively, Table 2). In turn, the ED_50_ for **KA-232** was very similar to the ED_50_ for the chemical prototype, **KA-11** (Table 2). Thus, in sum, the proposed structural modifications did not improve the protection of **KA-228** and **KA-232** in the *sc*PTZ model vs. **KA-11**. Nevertheless, both new compounds were effective in this test. The results obtained suggest that further chemical optimizations may hopefully lead to the identification of substances with improved activity in the *sc*PTZ model.

**Table 2:** Antiseizure and acute adverse effects of **KA-228** and **KA-232** in the seizure models and the chimney test in mice after i.p. administration.

Compound	ED_50_ MES (mg/kg) ± SD	ED_50_ 6 Hz (mg/kg) ± SD	ED_50_ PTZ (mg/kg) ± SD	TD_50_ (mg/kg) ± SD	TI
**KA-11** [6]	88.4 ± 34.16	21.0 ± 32.19	59.9 ± 19.55	>1500	>16.97 (MES) >71.43 (6 Hz) >25.04 (PTZ)
**KA-228**	92.5 ± 16.00	76.4 ± 40.76 ****	95.4 ± 39.78 *	131.40 ± 36.24	1.42 (MES) 1.72 (6 Hz) 1.38 (PTZ)
**KA-232**	61.8 ± 26.94 **	26.5 ± 33.46	59.3 ± 78.19	162.80 ± 31.20	2.63 (MES) 6.14 (6 Hz) 2.75 (PTZ)
**VPA** [8,19]	355.2 ± 108.1	110.5 ± 42.8	176.2 ± 81.8	430.7 ± 47.1	1.21 (MES) 3.90 (6 Hz) 2.44 (PTZ)

Results are ED_50_ (± SD) values of **KA-228** and **KA-232** that protected 50% of the mice from MES, 6 Hz, PTZ-induced seizures. * *p* < 0.05, ** *p* < 0.01, **** *p* < 0.0001 vs. **KA-11**-treated animals (one-way ANOVA and post hoc Dunnett’s test). The results for **KA-11** were used from Kamiński et al. [6] (see compound **11**). TI, Therapeutic Index (TD_50_/ED_50_). The results for **KA-11** were used from Kaminski et al. [6]; see compound **11**. ED_50_ and TD_50_ of valproic acid (VPA) were used from Zagaja et al. [8,19] and Kaminski et al. [20].

**Figure 7:**
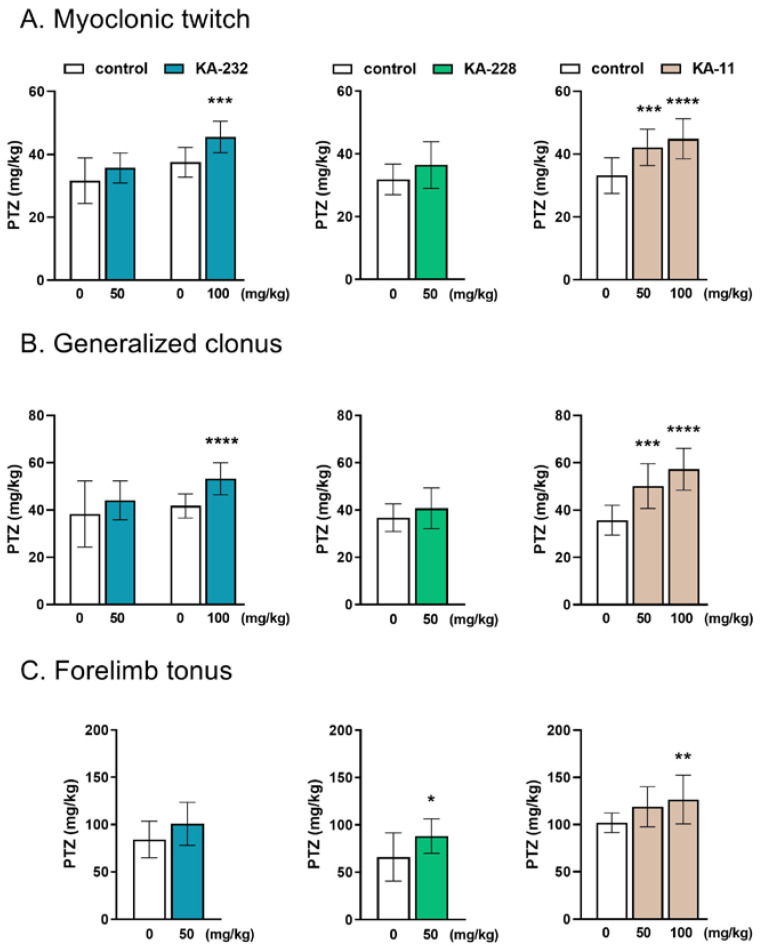
Effects of **KA-11**, **KA-228** and **KA-232** on the threshold for the first myoclonic twitch (**A**), generalized clonus (**B**) and forelimb tonus (**C**) in the *iv*PTZ test in mice. **KA-228** and **KA-232** were administered i.p., 30 min before seizure induction. Control animals received vehicle. Data are presented as means ± SD (mg/kg PTZ; *n* = 9–14 animals; the *n* value varies between groups because some animals were excluded from the study due to unsuccessful catheterization of the vein). The statistical significance for **KA-228** and **KA-232** was evaluated by a Student’s *t* test. Data for **KA-11** were used from Socała et al. [17] and re-analyzed using one-way ANOVA followed by Dunnett’s post hoc test. * *p* < 0.05, ** *p*  <  0.01, *** *p*  <  0.001, **** *p*  <  0.0001 vs. the vehicle-treated group (GraphPad Prism 8).

The *iv*PTZ test was used to further characterize the effect of **KA-228** and **KA-232** on seizure susceptibility in mice. As shown in Figure 7, **KA-228** administered at a dose of 50 mg/kg significantly raised the threshold for the tonic extension of forelimbs. However, it did not cause any significant changes in the thresholds for the first myoclonic twitch and generalized clonus with the loss of the righting reflex. Additionally, due to the unfavorable ED_50_ results in the above-mentioned acute seizure models (MES, 6 Hz, *sc*PTZ), the **KA-228** analog was not tested in the *iv*PTZ test at a dose of 100 mg/kg.

**KA-232** injected at a dose of 50 mg/kg did not significantly affect the thresholds for the onset of all three studied endpoints; however, at a dose of 100 mg/kg, it slightly raised the thresholds for myoclonic and clonic seizures (Student’s *t* test: *p* < 0.001 and *p* < 0.0001, respectively).

In the PTZ-induced seizure models, clonic seizures are followed by the tonic extension of the forelimb. Forelimb tonus is usually (but not always) followed by the tonic extension of hindlimbs and death caused by respiratory arrest [21]. Interestingly, hindlimb tonus did not occur in 5 out of 9 mice treated with **KA-232** at 50 mg/kg (Fisher’s exact test: *p* < 0.01 vs. control group), whereas **KA-232** at 100 mg/kg completely abolished the forelimb tonus in 7 out of 11 mice (Fisher’s exact test: *p* < 0.01 vs. control group). In the four remaining mice, the forelimb tonus was not followed by hindlimb tonus.

Referring to the results for **KA-11** (100 mg/kg) obtained by Socała and coworkers [22], which indicated a significant increase in the thresholds for all of the studied endpoints (myoclonic twitch, generalized clonus and forelimb tonus), we can conclude that the modifications did not result in a more favorable antiseizure efficiency for **KA-228** and **KA-232**.

To assess the safety profile and potential acute adverse effects of the studied compounds, we performed a chimney test evaluating motor coordination in mice (see TD_50_ values in Table 2). The obtained results indicated that both **KA-228** and **KA-232** analogs, unlike **KA-11** [6], disturbed motor performance in mice (131.40 ± 36.24, 162.80 ± 31.20 vs. >1500, respectively). In turn, the therapeutic index (TI) value in all models of epilepsy was more favorable for the **KA-232** than for the **KA-228** analog. Bearing in mind that the TI for an effective and safe drug should be higher than 2 [8], **KA-232** with TIs of 2.63 (MES test), 6.14 (6 Hz, 32 mA test) and 2.75 (*sc*PTZ test) is worth further advanced preclinical research (including subsequent structural modifications), especially since VPA, the most common first-line drug used in epilepsy, revealed distinctly lower TI values, especially in the MES and the 6 Hz (32 mA) seizure models [8].

**Figure 8:**
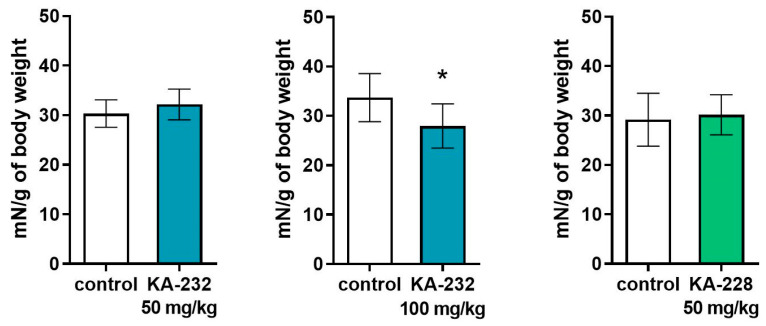
Effects of **KA-228** and **KA-232** on neuromuscular strength in mice. **KA-228** and **KA-232** were administered i.p., 30 min before the test. Control animals received vehicle. Data are presented as mean ± SD grip strengths in millinewtons per gram of mouse body weight (mN/g); *n* = 10 animals. The statistical significance was evaluated using the Student’s *t* test: * *p* < 0.05 vs. the vehicle-treated group (GraphPad Prism 8).

**KA-228** and **KA-232** at a dose of 50 mg/kg did not produce any significant effects on the neuromuscular strength measured in the grip strength test. Similarly, no statistically significant abnormalities in muscle strength in mice were observed for **KA-11** after a single administration at a dose of 30 mg/kg by Zagaja et al. [8]. However, **KA-232** at a dose of 100 mg/kg slightly decreased the grip strength (Student’s *t* test: *p* < 0.05, Figure 8). Thus, it appears that **KA-232** at higher doses may produce acute adverse effects such as impairment of neuromuscular strength.

In sum, on the basis of the above in vivo data, it can be concluded that compound **KA-232**, which was designed as a water-soluble derivative of the leading compound **KA-11**, offers a wide spectrum of antiseizure activities, as well as a satisfying safety profile (nevertheless, not better than **KA-11**). Certainly, the greatest advantage of **KA-232** compared to **KA-11** is water solubility, and thus better and faster absorption (bioavailability), and in turn expected improved effectiveness. When responding to seizures immediately, such drugs, especially if their mechanism of action is known, have a great chance to quickly stop seizures. However, this rapid and more effective absorption, as well as its potentially higher concentration in the brain may also reflect undesirable consequences, including an increased motor dysfunction, resulting from, e.g., interaction with off-targets. Nevertheless, a better understanding of this issue certainly requires a more detailed pharmacokinetic/pharmacodynamics analysis.

### 2.4. In Vitro Binding Assays

Dysfunction of both sodium and calcium ion channels is known to be one of the crucial reasons behind the pathogenesis of seizures [23,24]. Consequently, these ion channels are molecular targets for several clinically important ASDs such as lacosamide, lamotrigine, carbamazepine, oxcarbazepine and compounds currently under preclinical and clinical studies [25]. Thus, for **KA-228** and **KA-232**, we tested herein their binding profile toward the aforementioned ion channels at concentrations of 10 and 100 µM (Table 3). Notably, **KA-232**, which revealed potent activity in the in vivo seizure models, was also evaluated in a panel of additional assays including ionotropic or metabotropic receptors involved in the mechanism of action of known ASDs (i.a., GABA_A_, AMPA, NMDA, etc.) at a concentration of 10 µM. Apart from in vitro studies focused on explaining the mechanism of action, **KA-232** was additionally tested for its interaction with the potassium channel (hERG) which is recognized as one of the most important ‘off-targets’ responsible for harmful arrhythmogenic activity. Thus, the interaction of potential drug candidates with hERG must be excluded at an early stage of development.

As presented in Table 3, both new compounds showed only moderate binding to the sodium (site 2) channel (**KA-232**) or Cav_1.2_ channel (**KA-228**) at the high concentration of 100 µM; however, they were devoid of activity at the lower concentration of 10 µM. Furthermore, a more comprehensive in vitro characterization of **KA-232** revealed that this molecule did not interact with other molecular targets for ASDs, namely NMDA, AMPA, GABA_A_, 5-HT_1A_ receptors, the Cav_2.2_ channel and the hERG channel (as ‘off-target’). Notably, the latter result indicates a low risk of the proarrhythmic activity of **KA-232**. As the binding studies were carried out at higher and lower concentrations (100 and 10 µM, respectively), it seems unlikely that the antiseizure activity of the **KA-232** compound, as well as the model compound **KA-11** (tested at 100 µM), is mediated by influence on any of the ion channels or receptors tested. Furthermore, our studies on a series of structurally related compounds exclude with high probability the influence of **KA-228** and **KA-232** on the GAT-1 transporter for GABA, Cav_3.2_ channels and SV2A protein (not tested herein) [7,9,26,27,28,29,30]. Bearing in mind that all compounds, namely **KA-228**, **KA-232** and the parent molecule **KA-11**, contain in their structures a phenylpiperazine fragment, which is one of the best-known pharmacophores for serotoninergic receptor (5-HTxR) ligands [6], it seems possible that antiseizure activity may be mediated by their influence on serotoninergic neurotransmission in the CNS. The serotoninergic mechanism of action is characteristic for selected ASDs or ASD candidates, such as naluzotan (5-HT_1A_R agonist) or lorcaserin (5-HT_2C_R agonist) [25]. Nevertheless, the obtained 5-HT_1A_R binding results (Table 3) showed no interaction for **KA-232**, nor its chemical prototype **KA-11**, at 10 µM and 100 µM, respectively. Further in vitro studies also proved that **KA-232** did not inhibit the TRPV1 channel at 10 µM in an antagonist functional assay. It should be stressed herein that according to the subject’s literature [31], the transient receptor potential cation channel, subfamily V member 1 (TRPV1), which is a nonselective (Ca^2+^ and Na^+^) cation channel, is involved in pain, psychiatric and epileptic disorders. Thus, in sum, all the in vitro assays performed for **KA-232** (and **KA-228**) did not enable the identification of a convincing mechanism of action responsible for their antiseizure activity. Therefore, in our opinion, more detailed and especially functional studies (i.e., electrophysiology) are necessary to elucidate the precise pharmacodynamics of **KA-228**, **KA-232** and **KA-11**.

### 2.5. In Vitro Absorption Distribution Metabolism Excretion and Toxicity (ADME-Tox) Studies

Taking into account the in vitro binding assays indicating more beneficial results for **KA-232**, the selected ADME-Tox parameters of this analog were evaluated in vitro and compared to the data obtained previously for **KA-11** [6]. The results are presented in Table 4.

In general, both compound **KA-232** and parent molecule **KA-11** showed satisfying and comparable drug-like properties. The high permeability of **KA-232** was sustained. The calculated Pe value (10.20 × 10^−6^ cm/s) was actually lower than that estimated for its derivative **KA-11** but similar to the highly permeable reference caffeine (10.44 × 10^−6^) (Table 4). However, after 5 h of incubation in a PAMPA plate, some products of **KA-232** degradation at the pyrrolidine-2,5-dione moiety were observed (see Supporting Material Figures S1 and S2).

Around 73% of **KA-232** remained after 120 min of incubation with HLMs (Table 5). This result is similar to the data obtained previously for **KA-11**. Three main metabolites were identified as the products of hydroxylation and/or the degradation/oxidation of pyrrolidine-2,5-dione. Moreover, the observed products of **KA-232** degradation in a physiological buffer in the PAMPA assay were also confirmed (see Supporting Material Figures S3–S7).

**Figure 9:**
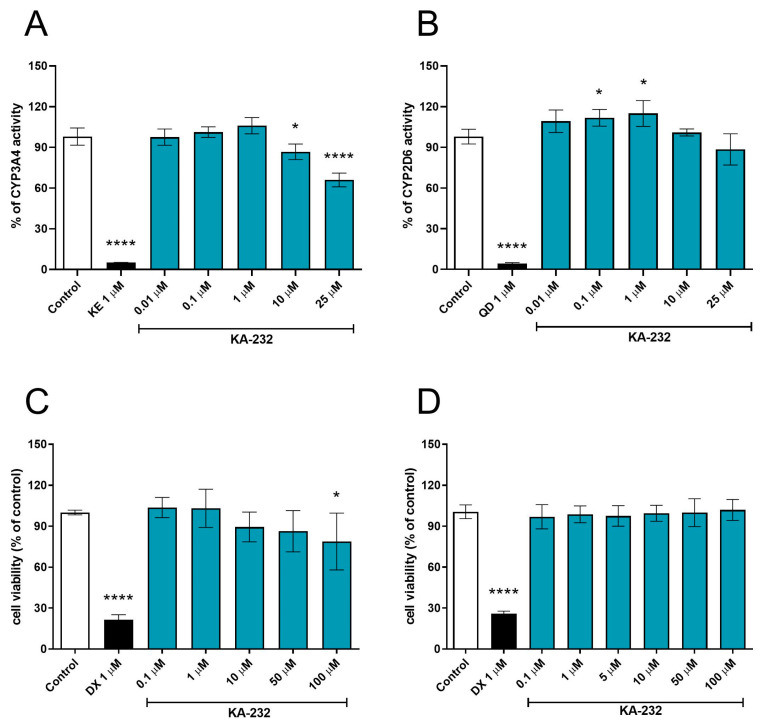
The effect of **KA-232** and the reference drug ketoconazole (KE) on CYP3A4 activity (**A**). The effect of **KA-232** and the reference drug quinidine (QD) on CYP2D6 activity (**B**). The effect of **KA-232** and the reference cytostatic drug doxorubicin (DX) on the HepG2 cell line (**C**) and the SH-SY5Y cell line (**D**) viability. Statistical significance (* *p* < 0.05, **** *p* < 0.0001) was analyzed by Graph Pad Prism 8 software using one-way ANOVA and Bonferroni’s Multiple Comparison Post Test.

Both compounds showed similar, slight effects on CYP3A4 activity only at the highest used doses of 10 and 25 µM. Similarly, a slight induction of CYP2D6 activity was observed: for **KA-11**, it was at concentrations of 1 and 10 µM, whereas for **KA-232**, it was at 0.1 and 1 µM (Figure 9A,B) (Table 4).

The statistically significant hepatotoxic effect was determined for both compounds only at the highest used dose of 100 µM. Moreover, the decrease in HepG2 cell viability in the presence of 100 µM of **KA-232** was approximately 78% of control, whereas at the 100× lower concentration of the reference cytostatic drug doxorubicin, this effect was determined as only 21% of control (Figure 9C). Interestingly, no neurotoxic effect of **KA-232** on neuroblastoma SH-SY5Y cell line was noted at all used doses (Figure 9D) (Table 4).

Overall, the ADME-Tox studies revealed that **KA-232** possesses a favorable membrane permeability and a satisfying safety profile in the hepatotoxicity, neurotoxicity and CYPS inhibition/induction studies. These in vitro results together with the potent in vivo antiseizure activity shown in the MES test may justify further and more detailed development of new, more potent and/or safer **KA-232** analogs.

## 3. Materials and Methods

### 3.1. Chemistry

All chemicals and solvents were purchased from commercial suppliers and were used without further purification. Melting points (mp.) were determined in open capillaries on a Büchi 353 melting point apparatus (Büchi Labortechnik, Flawil, Switzerland). Thin-layer chromatography (TLC) and gradient ultra-performance liquid chromatography (UPLC) were used to assess the purity and homogeneity of the compounds. TLC was carried out on silica gel 60 F_254_ pre-coated aluminum sheets (Macherey-Nagel, Düren, Germany), using the following developing systems: (S_1_–Dichloromethane methanol DCM:MeOH (9:0.3; *v*/*v*), S_2_–DCM:MeOH (9:0.5; *v*/*v*)). Spot detection: UV light (λ = 254 nm). UPLC and liquid chromatography–mass spectrometry (LC-MS) were obtained on Waters ACQUITY™ TQD system (Waters, Milford, CT, USA) with the MS-TQ detector and UV-Vis-DAD eλ detector. The ACQUITY UPLC BEH C18, 1.7 μm (2.1 × 100 mm), column was used with VanGuard Acquity UPLC BEH C18, 1.7 μm (2.1 × 5 mm) (Waters, Milford, CT, USA). Standard solutions (1 mg/mL) of each compound were prepared in analytical-grade acetonitrile (MeCN)/water mixture (1:1; *v*/*v*). Conditions applied were as follows: eluent A (water/0.1% HCOOH), eluent B (MeCN/0.1% HCOOH), a flow rate of 0.3 mL/min, a gradient of 5–100% B over 10 min and an injection volume of 10 μL. The UPLC retention times (t_R_) are given in min. Preparative column chromatography was performed using silica gel 60 (particle size 0.063–0.200; 70–230 Mesh ATM) purchased from Merck (Darmstadt, Germany). Elemental analyses (C, H and N) for final compounds were carried out by a micro method using the elemental Vario EI III Elemental analyzer (Hanau, Germany). The results of elemental analyses were within ±0.4% of the theoretical values. Proton nuclear magnetic resonance (^1^H NMR) and carbon nuclear magnetic resonance (^13^C NMR) spectra were obtained in a JEOL-500 spectrometer (JEOL USA, Inc., Peabody, MA, USA), in CDCl_3_ operating at 500 MHz (^1^H NMR) and 126 MHz (^13^C NMR). Chemical shifts are reported in δ values (ppm) relative to TMS δ = 0 (1H), as internal standard. The *J* values are expressed in Hertz (Hz). Signal multiplicities are represented by the following abbreviations: s (singlet), br s (broad singlet), d (doublet), dd (double doublet), ddd (double double doublet), dt (doublet of triplets), t (triplet), q (quartet), quin (quintet), m (multiplet).

#### 3.1.1. Synthetic Procedure for Boc-Protected Compound (Tert-butyl-(1-oxo-1-(4-(3-(trifluoromethyl)phenyl)piperazin-1-yl)propan-2-yl)carbamate (1))

To the DCM (100 mL) solution of *N*-(tert-butoxycarbonyl)-DL-alanine (5 g, 26.4 mmol, 1 eq) was successively added DCC (8.18 g, 39.6 mmol, 1.5 eq) dissolved in 15 mL of DCM. After stirring (15 min), the 1-(3-(trifluoromethyl)phenyl)piperazine (6.08 g, 26.4 mmol, 1.0 eq) was added dropwise, and the reaction was stirred at room temperature for 2 h. The DCM was evaporated in vacuo, and the product was purified by column chromatography using a DCM:MeOH–9:0.3 (*v*/*v*) mixture as a solvent system.

Tert-butyl-(1-oxo-1-(4-(3-(trifluoromethyl)phenyl)piperazin-1-yl)propan-2-yl)carbamate (1).

Light oil. Yield: 86% (9.12 g); TLC: R_f_ = 0.70 (S_1_); UPLC (purity > 99%): t_R_ = 7.32 min. C_19_H_26_F_3_N_3_O_3_ (401.43). LC-MS (ESI): *m*/*z* calcd for C_19_H_26_F_3_N_3_O_3_ (M+H)^+^ 401.19, found 402.2.

#### 3.1.2. Synthetic Procedure for Amine (2-Amino-1-(4-(3-(trifluoromethyl)phenyl)piperazin-1-yl)propan-1-one (2))

The DCM (30 mL) solution of 1 (9.0 g, 22.4 mmol, 1 eq) was treated with TFA (7.69 g, 5.00 mL, 67.2 mmol, 3 eq) and stirred at room temperature for 6 h. Next, DCM was evaporated in vacuo. The oil residue obtained was suspended in water (30 mL), and 25% ammonium hydroxide was carefully added to pH = 8. The aqueous phase was extracted using DCM (3 × 50 mL), dried over anhydrous Na_2_SO_4_ and concentrated to dryness. Compound 2 was obtained as an oil and used for further reactions without purification.

2-Amino-1-(4-(3-(trifluoromethyl)phenyl)piperazin-1-yl)propan-1-one (2). Light oil. Yield: 96% (6.5 g); UPLC (purity > 99%): t_R_ = 4.23 min. C_14_H_18_F_3_N_3_O (301.31). LC-MS (ESI): *m*/*z* calcd for C_14_H_18_F_3_N_3_O (M+H)^+^ 302.14, found 302.3.

#### 3.1.3. Synthetic Procedure for Unsaturated Maleiamic Acid (4-Oxo-4-((1-oxo-1-(4-(3-(trifluoromethyl)phenyl)piperazin-1-yl)propan-2-yl)amino)but-2-enoic acid (3))

Maleic anhydride (0.98 g 10.0 mmol, 1 eq) was added to a solution of 2 (3.00 g, 10.0 mmol, 1 eq) in AcOEt (100 mL) and stirred for 4 h. After this time, the solvent was distilled off to dryness. The compound was obtained as solid after washing with diethyl ether (Et_2_O).

4-Oxo-4-((1-oxo-1-(4-(3-(trifluoromethyl)phenyl)piperazin-1-yl)propan-2-yl)amino)but-2-enoic acid (3). White solid. Yield: 81% (3.22 g); UPLC (purity 96%): t_R_ = 5.82 min. C_18_H_20_F_3_N_3_O_4_ (399.37). LC-MS (ESI): *m*/*z* calcd for C_18_H_20_F_3_N_3_O_4_ (M+H)^+^ 400.14, found 400.2.

#### 3.1.4. Synthetic Procedure for Unsaturated Pyrrolidine-2,5-Dione Derivative (1-(1-Oxo-1-(4-(3-(trifluoromethyl)phenyl)piperazin-1-yl)propan-2-yl)-1H-pyrrole-2,5-dione (4))

ZnCl_2_ (1.05 g, 7.8 mmol, 1 eq) was added to the suspension of 3 (3.10 g, 7.8 mmol, 1 eq) in dry 1,4-dioxane (100 mL), and the mixture was heated to 80 °C. Then, solution of HMDS (2.41 g, 11.7 mmol, 1.5 eq) was added dropwise over 30 min. The reaction was continued with stirring in reflux for about 8 h, then cooled and concentrated under reduced pressure. After distilling off the solvent, the oily residue was dissolved in DCM and extracted with 0.1 M HCl (3 × 50 mL), water (3 × 50 mL) and saturated NaCl solution (3 × 50 mL). The organic layer was dried over anhydrous Na_2_SO_4_ and then evaporated to dryness. The crude product was purified by column chromatography. The compound was obtained as solid after washing with Et_2_O.

1-(1-Oxo-1-(4-(3-(trifluoromethyl)phenyl)piperazin-1-yl)propan-2-yl)-1H-pyrrole-2,5-dione (4). White solid. Yield: 80% (2.37 g); TLC: R_f_ = 0.42 (S_2_); UPLC (purity > 99%) t_R_ = 6.41 min. C_18_H_18_F_3_N_3_O_3_ (381.36). LC-MS (ESI): *m*/*z* calcd for C_18_H_18_F_3_N_3_O_3_ (M+H)^+^ 382.13 found 382.2.

#### 3.1.5. Synthetic Procedure for Target Water-Soluble Compound 3-(Dimethylamino)-1-(1-oxo-1-(4-(3-(trifluoromethyl)phenyl)piperazin-1-yl)propan-2-yl)pyrrolidine-2,5-dione hydrochloride (**KA-232**)

The dimethylamine solution in tetrahydrofuran (THF, 6.1 mmol, 1 eq) was added to a solution of 4 (2.30 g, 6.1 mmol, 1 eq) in dry benzene (80 mL). The reaction was continued with stirring for about 8 h, then concentrated under reduced pressure. The crude product was purified by column chromatography. The compound was then converted into hydrochloride salt by treating the compound with a 2M methanolic hydrochloric acid solution. The compound was obtained as solid after washing with Et_2_O.

3-(Dimethylamino)-1-(1-oxo-1-(4-(3-(trifluoromethyl)phenyl)piperazin-1-yl)propan-2-yl)pyrrolidine-2,5-dione hydrochloride (**KA-232**). White solid. Yield: 84% (2.16 g); mp. 141.4–143.5 °C; TLC: R_f_ = 0.55 (S_2_); UPLC (purity: >99%): t_R_ = 4.91 min. LC-MS (ESI): *m*/*z* calcd for C_20_H_25_F_3_N_4_O_3_ (M+H)^+^ (426.19), found 427.3. ^1^H NMR (500 MHz, DMSO-d_6_) δ 1.38–1.50 (m, 3H), 2.83 (s, 6H), 3.06–3.10 (m, 1H), 3.16 (br s, 1H), 3.21–3.32 (m, 4H), 3.43–3.51 (m, 3H), 3.61 (br s, 1H), 4.64–4.73 (m, 1H), 4.96–5.14 (m, 1H), 7.08 (d, *J* = 7.3 Hz, 1H), 7.18 (br s, 1H), 7.22 (dd, *J* = 7.7, 4.6 Hz, 2H), 7.40 (t, *J* = 8.0 Hz, 1H), 11.91–12.20 (m, 1H). For free base ^1^H NMR (500 MHz, CDCl_3_) δ 1.64 (dd, *J* = 7.3, 1.6 Hz, 3H), 2.39 (d, *J* = 10.5 Hz, 6H), 2.59–2.72 (m, 1 H), 2.82 (dt, *J* = 18.3, 9.0 Hz, 1H), 3.20 (br s, 4H), 3.43–3.73 (m, 3H), 3.81 (dd, *J* = 9.0, 5.2 Hz, 2H), 5.01 (quin, *J* = 7.4 Hz, 1H), 7.00–7.16 (m, 3H), 7.35 (t, *J* = 8.0 Hz, 1H). ^13^C NMR (126 MHz, CDCl_3_) δ 15.1, 31.0, 41.5 (d, *J* = 1.1 Hz), 47.8, 53.5, 62.5, 113.0 (d, *J* = 3.9 Hz), 117.0 (d, *J* = 3.9 Hz), 119.5 124.2, (d, *J* = 272.5 Hz), 129.9, 131.7 (q, *J* = 31.9 Hz), 151.0, 167.35, 174.6, 176.1. Anal. calcd for C_20_H_26_ClF_3_N_4_O_3_ (462.90): C: 51.89, H: 5.66, N: 12.10; Found C: 51.97, H: 5.41, N: 12.34.

#### 3.1.6. Synthetic Procedure for 2-((4-Bromobutyl)amino)-1-(4-(3-(trifluoromethyl)phenyl)piperazin-1-yl)propan-1-one (5)

To the DCM (50 mL) solution of 4-bromobutanoic acid (1.67 g, 10.0 mmol, 1.0 eq), was successively added DCC (3.10 g, 15.0 mmol, 1.5 eq) dissolved in 15 mL of DCM. After stirring (15 min), intermediate compound **2** (3.00 g, 10.0 mmol, 1 eq) was added, and the reaction was stirred at room temperature for 2 h. The DCM was evaporated in vacuo, and the product was purified by column chromatography using a DCM:MeOH–9:0.3 (*v*/*v*) mixture as a solvent system.

2-((4-bromobutyl)amino)-1-(4-(3-(trifluoromethyl)phenyl)piperazin-1-yl)propan-1-one (5)

White solid. Yield: 85% (3.69 g); TLC: R_f_ = 0.51 (S_1_); UPLC (purity: 91%) t_R_ = 4.37 min. C_18_H_25_BrF_3_N_3_O(436.32). LC-MS (ESI): *m*/*z* calcd for C_18_H_25_BrF_3_N_3_O (M+H)^+^ 437.1 found 437.6.

#### 3.1.7. Synthetic Procedure for Pyrrolidin-2-One Derivative 1-(1-Oxo-1-(4-(3-(trifluoromethyl)phenyl)piperazin-1-yl)propan-2-yl)pyrrolidin-2-one (**KA-228**)

NaH (0.40 g, 16.5 mmol, 2 eq) was added to a solution of 5 (3.60 g, 8.25 mmol, 1 eq) in anhydrous THF. The reaction mixture was stirred for 3 h and next concentrated under reduced pressure. The oily residue was dissolved in 0.1 M HCl (50 mL) and extracted with DCM (3 × 50 mL). The organic layer was dried over anhydrous Na_2_SO_4_ and evaporated to dryness. The crude product was purified by column chromatography using DCM:MeOH (9:0.5; *v*/*v*) solvent system. After washing with Et_2_O, the compound was obtained as a white solid.

1-(1-oxo-1-(4-(3-(trifluoromethyl)phenyl)piperazin-1-yl)propan-2-yl)pyrrolidin-2-one (**KA-228**)

White solid. Yield: 88% (2.68 g); mp. 87.1–88.4 °C; TLC: R_f_ = 0.48 (S_2_); UPLC (purity: >99%): t_R_ = 5.82 min. LC-MS (ESI): *m*/*z* calcd for C_18_H_22_F_3_N_3_O_2_ (M+H)^+^ (370.17), found 370.3. ^1^H NMR (500 MHz, CDCl_3_) δ 1.32 (d, *J* = 6.9 Hz, 3H), 1.96–2.09 (m, 2H), 2.28–2.48 (m, 2H), 2.96–3.17 (m, 2H), 3.25 (ddd, *J* = 8.8, 6.7, 3.0 Hz, 2H), 3.32–3.51 (m, 2H), 3.59–3.72 (m, 2H), 3.73–3.97 (m, 2H), 5.16 (q, *J* = 6.9 Hz, 1H), 7.03 (dd, *J* = 8.3, 2.3 Hz, 1H), 7.07 (s, 1H), 7.10 (d, *J* = 7.6 Hz, 1H), 7.35 (t, *J* = 8.0 Hz, 1H). ^13^C NMR (126 MHz, CDCl_3_) δ 14.8, 18.2, 31.0, 41.9, 43.3, 45.2, 46.1, 48.9, 49.5, 112.7 (q, *J* = 3.9 Hz), 116.8 (d, *J* = 3.8 Hz), 119.3 (d, *J* = 1.2 Hz), 124.2 (q, *J* = 272.4 Hz), 129.8, 131.7 (q, *J* = 31.8 Hz), 150.6, 169.1, 174.3. Anal. calcd for C1_8_H_22_F_3_N_3_O_2_ (369.39): C: 58.53, H: 6.00, N: 11.38; Found C: 58.97, H: 6.11, N: 11.45.

### 3.2. In Silico Determination of Selected Physicochemical Descriptors

Physicochemical properties of **KA-228** and **KA-232** and BOILED-Egg predictive model were determined using the online tool SwissADME website [14].

### 3.3. In Vivo Studies

#### 3.3.1. Animals and Experimental Conditions

The experiments were conducted on adult male albino Swiss mice weighing 22–26 g. The animals were kept in colony cages under standardized laboratory conditions: natural light–dark cycle 12/12 h, temperature 20–24 °C, air humidity 45–65% and free access to tap water and food (chow pellets). After 7 days of adaptation to laboratory conditions, the animals were randomly assigned to experimental groups consisting of 8 mice. The experimental procedures described herein were approved by the Local Ethics Committee in Lublin, Poland (approval numbers: 71/2020 and 13/2021), and were conducted in accordance with EU Directive 2010/63/EU for animal experiments. All efforts were made to minimize animal suffering and to use only the number of animals necessary to produce reliable scientific data according to the 3Rs rule [32].

#### 3.3.2. Drugs

The following drugs were used: **KA-228** and **KA-232** (synthesized by the Department of Medicinal Chemistry from the Jagiellonian University Medical College, Faculty of Pharmacy); pentylenetetrazole, PTZ (Sigma-Aldrich, Saint Louis, MO, USA); 0.5% solution of tetracaine hydrochloride (Sigma-Aldrich, Saint Louis, MO, USA).

All substances (except PTZ and tetracaine hydrochloride dissolved in water for injections) were suspended in a 1% solution of Tween 80 (Sigma-Aldrich, Saint Louis, MO, USA) in water for injections (Baxter, Warszawa, Poland) or 0.9% NaCl. All drugs were injected intraperitoneally (i.p.) 30 min before all seizure models with 1 mL syringes as a single injection, in a volume of 10 mL/kg. The pretreatment time (30 min) before testing for **KA-228** and **KA-232** was chosen based on the time to peak of maximum anticonvulsant activity of **KA-11** from our previous studies [6,8,22].

All reference drugs used in ADME-Tox in vitro assays (caffeine, ketoconazole, quinidine and doxorubicin) were purchased from Sigma-Aldrich (St. Louis, MO, USA) and dissolved in DMSO (10 mM).

#### 3.3.3. Maximal Electroshock Seizure (MES) Test

The tonic–clonic seizures in mice were evoked by an electric stimulus (an alternating current 25 mA, 50 Hz, maximum output voltage 500 V, 0.2 s) generated by a rodent shocker (Hugo Sachs Elektronik, Freiburg, Germany) and delivered via ear-clip electrodes. The animals were administered with increasing doses of **KA-228** and **KA-232**, and the anticonvulsant activity of each drug was evaluated as the ED_50_ value (median effective dose of the tested drug, which protects 50% of mice against seizures). This experimental procedure has been described in detail in our previous studies [33,34].

#### 3.3.4. 6-Hz (32 mA) Seizure Model

The 6-Hz-induced psychomotor seizures in mice were evoked by current (6 Hz, 0.2 ms rectangular pulse width, 32 mA, 3 s duration) generated by an S48 Square Pulse Stimulator and CCU1 Constant Current Unit (Grass Technologies, West Warwick, RI, USA). The animals were administered with increasing doses of **KA-228** and **KA-232**, and the anticonvulsant activity of each drug was evaluated as the ED_50_. This experimental procedure has been described in detail in our previous studies [19,35].

#### 3.3.5. scPTZ-Induced Convulsions

The PTZ-induced clonic seizures in mice were determined after *sc* administration of PTZ at a dose of 100 mg/kg. The animals were administered with increasing doses of **KA-228** and **KA-232**, and the anticonvulsant activity of each drug was evaluated as the ED_50_ value. This experimental procedure has been described in detail in our previous studies [20,36].

#### 3.3.6. Timed ivPTZ Seizure Threshold Test

The timed *iv*PTZ seizure threshold test was carried out according to the method described by Socała et al. [22]. **KA-228** and **KA-232** were suspended in a 1% solution of Tween 80 and administered i.p. 30 min before the test. In the experiment with **KA-232**, two separate control groups were used because the effects of **KA-232** at a dose of 50 and 100 mg/kg were determined on different days.

#### 3.3.7. Behavioral Tests

##### Chimney Test

The chimney test of Boissier et al. [37] was used to quantify the acute adverse-effect potential of **KA-228** and **KA-232** on motor performance in mice. The acute neurotoxic effects of **KA-228** and **KA-232** were expressed as median toxic doses (TD_50_ values in mg/kg), at which these substances impaired motor coordination and balance in 50% of the tested mice. This experimental procedure has been described in detail in our previous studies [30,31].

##### Grip Strength Test

The grip strength test was carried out according to the methodology described by Socała et al. [22]. Neuromuscular strength was measured just before the *iv*PTZ seizure threshold test to limit the number of animals used.

### 3.4. In Vitro Binding Assays

Binding studies were carried out commercially in Eurofins Laboratories (Poitiers, France or New Taipei City, Taiwan). The experimental conditions and references are listed in Table 5 and Table 6.

### 3.5. In Vitro ADME-Tox Studies

The assays and protocols used for evaluating the **KA-232** ADME-Tox parameters were similar to those described previously for compound **KA-11** [6]. Pre-coated PAMPA Plate System Gentest™ was purchased from Corning (Tewksbury, MA, USA). Human liver microsomes (HLMs) were provided by Sigma-Aldrich (St. Louis, MO, USA). The in silico studies of metabolism were performed with MetaSite 6.0.1 software (Molecular Discovery Ltd., Hertfordshire, UK). The influence on human cytochromes was estimated with use of CYP3A4 and CYP2D6 P450-Glo™ kits (Promega, Madison, WI, USA). Cell-based safety tests were performed with hepatoma HepG2 (HB-8065™) and neuroblastoma SH-SY5Y (CRL-2266™) cell lines obtained from ATCC^®^ (American Type Culture Collection, Manassas, VA, USA). The CellTiter 96^®^ AQueous Non-Radioactive Cell Proliferation Assay (Promega, Madison, WI, USA) was used for determination of cell viability. The luminescent signal and the absorbances were measured by using a microplate reader EnSpire PerkinElmer (Waltham, MA, USA). The LC/MS/MS analyses used in PAMPA and metabolic stability assays were obtained on Waters ACQUITY™ TQD system (Waters, Milford, CT, USA).

### 3.6. Statistical Analyses

The ED_50_ or TD_50_ values for tested substances in the acute animal models of seizures were calculated using the computer log-probit analysis [49]. Subsequently, the ED_50_ and TD_50_ values (±SEM) were statistically analyzed using one-way analysis of variance (ANOVA) followed by the post hoc Dunnett’s test for multiple comparisons. Differences among values were considered statistically significant if *p* < 0.05. GraphPad Prism version 8 for Windows (GraphPad Software, San Diego, CA, USA) was used as a statistical software program. The results obtained from the acute *iv*PTZ test and grip strength test were evaluated by a Student’s *t* test. Fisher’s exact probability test was used to analyze protection from tonic forelimb and hindlimb extension in the *iv*PTZ test.

## 4. Conclusions

The ‘me-too’ strategy is one of the most effective chemical tools in medicinal chemistry, which enables the discovery of new drug candidates by applying small structural modifications of the leading molecule. Thus, in the current studies, we proposed two targeted and rational structural modifications of the leading compound **KA-11**, with the aim of obtaining more effective or safer derivatives.

Based on the in vivo results, it may be concluded that both (**KA-228** and **KA-232**) modifications (within the imide ring) proved to be antiseizure active compounds; nevertheless, their efficacy and safety profile was not more favorable compared to parent **KA-11**. However, it should be stressed that **KA-232** (a water-soluble hydrochloride salt) was distinctly more potent in the MES test vs. **KA-11**, and therefore this compound should be considered for more extensive chemical and pharmacological development, including efficacy and safety assessment, as well as pharmacokinetic characterization.

In addition, the favorable results obtained for the leading compound **KA-11** (named C11) in the PILO-induced SE model in mice [11], showing strong protection of cognitive functions and stimulation of neurogenesis, certainly encourage the continuation and planning of more advanced studies for the parent **KA-11**, as well as the **KA-232** analog using an animal model of chronic epilepsy. Bearing in mind that **KA-11** as a hybrid molecule combines the structural fragment of levetiracetam, it would certainly be worth using the PILO model of temporal lobe epilepsy, where this ASD shows strong antiseizure effectiveness. These studies would provide valuable data on the anticonvulsant and neuroprotective effects of chronic treatment with **KA-11** and **KA-232**.

Extremely important in ASD treatment is that, depending on the type of the mechanism, as well as the multidirectional way of action, it can be used in various neurological diseases both with and without symptoms of seizures [50,51,52]. Thus, it seems reasonable to extend the preclinical development of compounds discovered by our team to other neurological or neurodegenerative conditions regardless of convulsive symptoms such as neuropathic pain, Alzheimer’s and Parkinson’s diseases, migraine headaches, bipolar disorders or anxiety. When developing new potential candidates for ASDs, we place a great emphasis on compounds having multiple molecular targets and the lack of side effects. Taking into account the long-term process of therapy, such drugs could be successfully used in other neurological diseases.

Undoubtedly, **KA-232**, due to its solubility in water, may be of great interest in further preclinical studies, such as models of status epilepticus, where the time and route (i.v.) of compound administration play a significant role in the antiseizure activity.

## Figures and Tables

**Table 1:** Drug-like parameters estimated according to Lipinski’s and Veber’s rules.

Compound	Lipinski Rule				Veber Rule		CNS MPO
	MW ^a^ ≤500	Log P ^b^ ≤5	HBD ^c^ ≤5	HBA ^d^ ≤10	NBR ^e^ ≤10	TPSA ^f^ ≤140 Å^2^	
**KA-232**	426.43	1.82	0	7	6	64.17	5.53
**KA-228**	369.38	2.41	0	5	5	43.86	5.93
**KA-11** [8]	383.37	1.98	0	6	5	60.93	5.83

^a^ MW: molecular weight; ^b^ Log P lipophilicity values; ^c^ HBD: hydrogen bond donors; ^d^ HBA: hydrogen bond acceptors; ^e^ NBR: number of rotatable bonds; ^f^ TPSA: topological polar surface area. Data for **KA-11** were used from Zagaja et al. [8].

**Table 3:** In vitro binding assays for **KA-228**, **KA-232** and chemical prototype **KA-11**.

Binding Studies	Source	% Inhibition of Control Specific Binding (Concentration (µM)) ^a^		
		KA-228	KA-232	KA-11
Na^+^ channel (site 2)	Rat cerebral cortex	15.5 (100) −2.9 (10)	26.2 (100) 3.8 (10)	37.4 (100) *
Calcium Cav_1.2_ channels (dihydropyridine site, antagonist radioligand)	Rat cerebral cortex	32.5 (100) 8.7 (10)	18.5 (100) −6.3 (10)	64.2 (100) *
Calcium Cav_1.2_ channels (diltiazem site, antagonist radioligand)	Rat cerebral cortex	NT	17.5 (100) −1.8 (10)	44.9 (100) ^#^
Calcium Cav_1.2_ channel (verapamil site, antagonist radioligand)	Rat cerebral cortex	NT	−2.8 (10)	1.5 (100) ^#^
Glutamate (NMDA, nonselective, antagonist radioligand, [3H] Ifenprodil radioligand)	Rat cerebral cortex	NT	15.4 (10)	13.0 (100) ^#^
Glutamate (AMPA, nonselective, antagonist radioligand, [3H] AMPA radioligand)	Rat cerebral cortex	NT	3.7 (10)	−2.9 (100) ^#^
Calcium Cav_2.2_ channel (antagonist radioligand)	Rat cerebral cortex	NT	10.6 (10)	13.9 (100) *
GABA_A_ (α_1_,β_2_,γ_2_) (agonist radioligand)	Human recombinant receptor	NT	−6.7 (10)	−28.1 (100) ^#^
5-HT_1A_ (agonist radioligand)	Human recombinant receptor	NT	9.6 (10)	19.5 (100) ^#^
Potassium channel (hERG)	Human recombinant HEK-293 cells	NT	18.3 (100)	15.0 (100) **
**Functional studies**	Source	% Inhibition of control specific binding (concentration (µM)) ^a^		
		**KA-228**	**KA-232**	**KA-11**
TRPV1 (VR1) (antagonist effect)	Human recombinant CHO cells.	NT	10.5 (10)	33.0 (100) **

^a^ Results showing activity higher than 50% are considered to represent significant effects of the tested compounds; results showing an inhibition between 25% and 50% are indicative of moderate effect; results showing an inhibition lower than 25% are not considered significant and mostly attributable to variability in the sign around the control level. Data for **KA-11** from * Kamiński [6] (see compound **11**), ^#^ Socała et al. [9]. ** not published before. NT—not tested.

**Table 4:** Results of the in vitro ADME-Tox studies for **KA-232** and chemical prototype **KA-11**.

ADME-Tox Assay In Vitro	KA-232	KA-11
PAMPA Pe (10^−6^ cm/s] ± SD	10.20 ± 1.65 ^a^	27.1 ± 3.8 ^b^ [9]
Metabolic stability (% remaining after incubation with HLMs)	73.81	73.49 [6]
CYP3A4 activity (% of control ± SD at 10 μM)	86.77 ± 5.71	99.21 ± 4.9 [6]
CYP2D6 activity (% of control ± SD at 10 μM)	100.96 ± 2.57	122.30 ± 2.02 [9]
HepG2 viability (% of control ± SD at 10 μM)	89.44 ± 10.22	99.78 ± 2.18 [9]
SH-SY5Y viability (% of control ± SD at 10 μM)	99.44 ± 5.89	not tested

^a^ The result for reference caffeine in performed parallel artificial membrane permeability (PAMPA) assay for **KA-11** = 15.1 ± 0.4 [9]. ^b^ The result for reference caffeine in performed PAMPA assay for **KA-232** = 10.44 ± 1.88.

**Table 5:** Experimental conditions used in in vitro binding assays.

Assay/Ion Channels	Source	Ligand	Conc. (nM)	K_d_ (nM)	Non-Specific	Incubation	Detection Method	Ref.
**Na^+^ channel** **(site 2) (antagonist radioligand)**	rat cerebral cortex	[^3^H]batracho- toxin	10	91	veratridine (300 µM)	60 min 37 °C	Scintillation counting	[38]
**Calcium Cav_1.2_ channel (dihydropyridine site) (antagonist radioligand)**	rat cerebral cortex	[^3^H]nitrendi- pine	0.25	0.27	nitrendipine (1 µM)	90 min RT	Scintillation counting	[39]
**Calcium Cav_1.2_ channel (diltiazem site) (benzothiazepines) (antagonist radioligand)**	rat cerebral cortex	[^3^H]diltiazem	15	52	diltiazem (10 µM)	120 min RT	Scintillation counting	[40]
**Calcium Cav_1.2_ channel (verapamil site, antagonist radioligand)**	rat cerebral cortex	[^3^H](-)- desmethoxy- verapamil	0.4	14	methoxyvera- pamil (10 µM)	60 min RT	Scintillation counting	[41]
**Glutamate (NMDA, nonselective, antagonist radioligand**	rat cerebral cortex	[^3^H]ifenprodil	2	26	ifenprodil (10 µM)	120 min 4 °C	Scintillation counting	[42]
**Glutamate (AMPA, nonselective, antagonist radioligand)**	rat cerebral cortex	[^3^H]AMPA	5	18	L-glutamic acid (1000 µM)	90 min 4 °C	Scintillation counting	[43]
**Calcium Cav_2.2_ channel (antagonist radioligand)**	rat cerebral cortex	[^125^I]ω-conotoxin GVIA	0.01	0.051	w-conotoxin GVIA (0.1 µM)	30 min 4 °C	Scintillation counting	[44]
**GABA_A_ (** **α** **_1_** **,** **β** **_2_** **,** **γ** **_2_** **) (agonist** **radioligand)**	human recombinant receptor	[^3^H]muscimol	15	30	muscimol (10 µM)	120 min RT	Scintillation counting	[45]
**5-HT_1A_ (agonist radioligand)**	human recombinant receptor	[^3^H]8-OH-DPAT	1.5	2	metergoline (10 µM)	60 min RT	Scintillation counting	[46]
**Potassium channel (hERG)**	human recombinant HEK-293 cells	[^3^H]dofetilide	3	6.6	dofetilide (10 µM)	60 min RT	Scintillation counting	[47]

**Table 6:** The experimental conditions used in in vitro functional assays.

Assay/Ion Channel	Source	Control Activator	Control Inhibitor	Measured Component	Incubation	Detection Method	Ref.
**TRPV1 (VR1)** ***(h)*** **(antagonist effect)**	human recombinant (CHO cells)	capsaicin (30 nM)	capsazepine	intracellular [Ca^2+^]	RT	Fluorimetry	[48]

## Data Availability

No new data were created.

## Supplementary Materials

The following supporting information can be downloaded at https://www.mdpi.com/article/10.3390/ijms24098302/s1.

## Abbreviations

ASD	antiseizure drug
MES test	maximal electroshock seizure test
6 Hz test	6 Hertz test
scPTZ test	subcutaneous pentylenetetrazole test
ivPTZ test	intravenous pentylenetetrazole test
ADME-Tox	Absorption Distribution Metabolism Elimination and Toxicology
PILO	pilocarpine
PILO-induced SE	pilocarpine induced status epilepticus
DCC	(N,N′-dicyclohexylcarbodiimide)
TFA	trifluoroacetic acid
HMDS	hexamethyldisilazane
MW	molecular weight
Log P	lipholicity values
HBD	hydrogen bond donors
HBA	hydrogen bond acceptors
NBR	number of rotatable bonds
TPSA	topological polar surface area
LIPO	lipophilicity
POLAR	polarity
INSOLU	solubility
FLEX	flexibility
INSATU	saturation of the molecule
CNS	central nervous system
MPO	multiparameter optimization
GI	gastrointestinal
HIA	human intestinal absorption
BBB	blood-brain barrier
PGP+/−	poliglicoprotein
ED50	effective dose
TD50	Toxicity dose
SD	Standard Deviation
TI	Therapeutic Index
VPA	valproic acid
i.p.	intraperitoneal injection
GABAA	γ-aminobutyric acid A
AMPA	α-amino-3-hydroxy-5-methyl-4-isoxazolepropionic acid
NMDA	N-methyl-D-aspartic acid
hERG	human ether-à-go-go-related gene
TRPV1	Transient receptor potential vanilloid 1
VR1	Vanilloid type 1 receptor
5-HT1A	serotonin 1A
GAT-1	GABA transporter 1
SV2A	synaptic Vesicle Glycoprotein 2A
PAMPA	parallel artificial membrane permeability
HLMs	Human liver microsomes
CYP3A4	Cytochrome P450 3A4
CYP2D6	Cytochrome P450 2D6
HepG2	Hepatoblastoma cell line
SH-SY5Y	Human Neuroblastoma cell line
