# Supplementary material for: In Vivo and In Vitro Characterization of Close Analogs of Compound KA-11, a New Antiseizure Drug Candidate
Source: Int J Mol Sci. 2023 May 5;24(9):8302. doi: 10.3390/ijms24098302 (PMC10179080; doi:10.3390/ijms24098302)
Supplement: Supplementary file 1 [file ijms-24-08302-s001.zip › ijms-2343302-supplementary.pdf]

Supporting information

1. PAMPA

Donor well

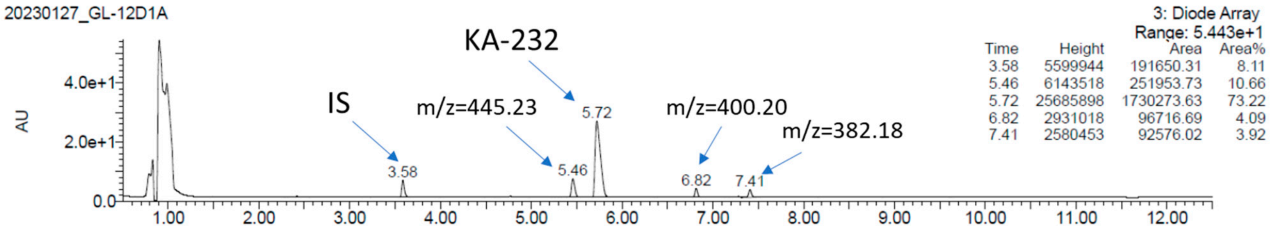

Acceptor well

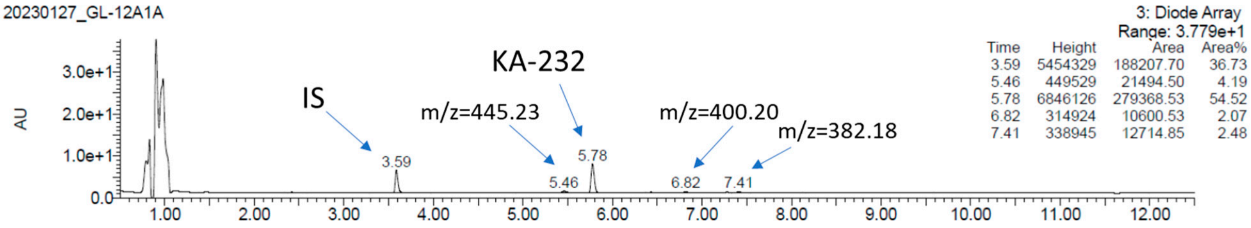

Figure S1 UPLC of **KA-232** solution in PBS (pH=7.4) after 5h of incubation in donor and acceptor well (RT). IS = internal standard. The products of compound's decomposition in PBS buffer were also found.

m/z=382.18:

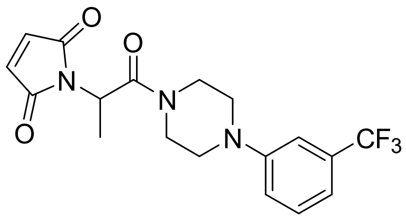

m/z=400.20:

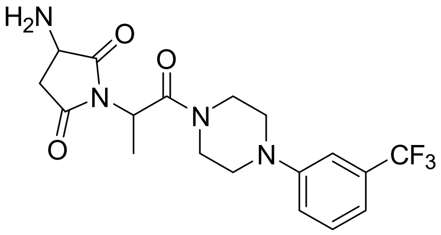

Figure S2 The proposed products of **KA-232** decomposition after incubation in PBS buffer (pH 7.4, RT, 5h). The compound with m/z=445.23 was determined as a hydrolyzed derivative.

2. Metabolic stability

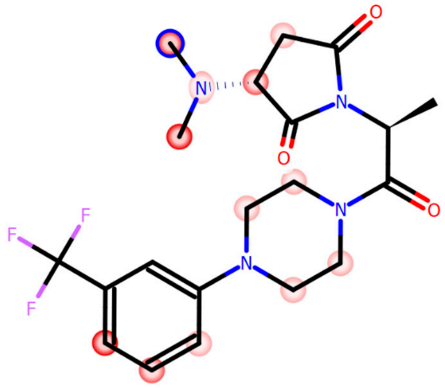

Figure S3 The MetaSite 6.0.1. software prediction of the most probably sites of **KA-232** metabolism. The darker red color - the higher probability to be involved in the metabolism pathway. The blue circle marked the site of compound with the highest probability of metabolic bioconversion.

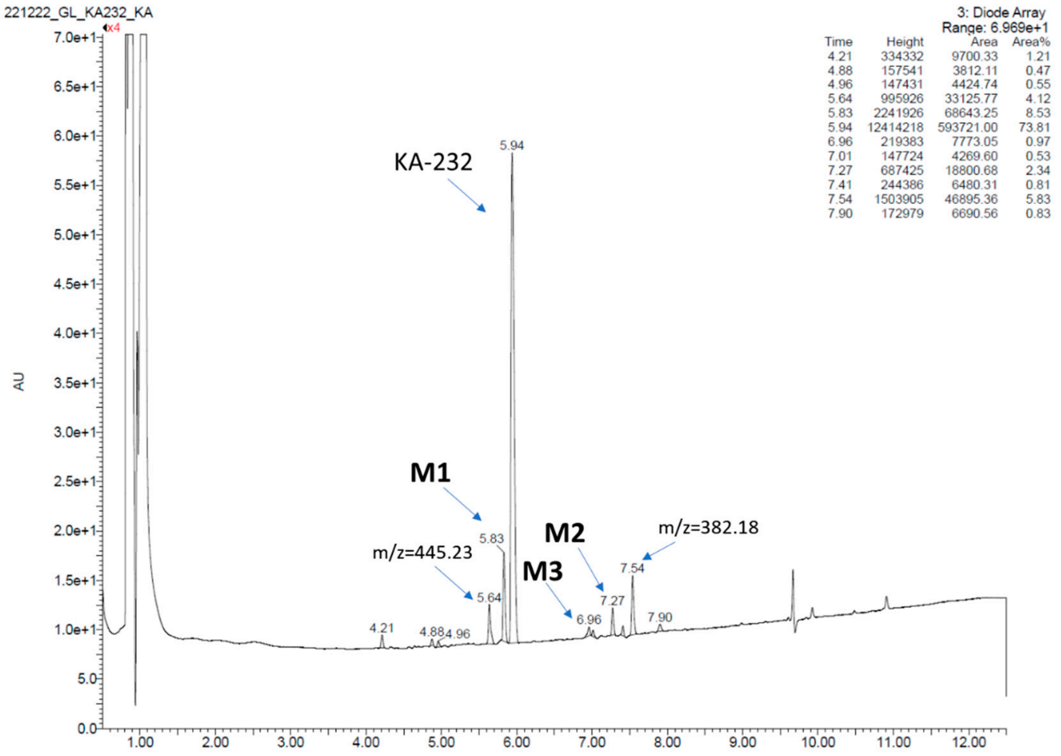

Figure S4 UPLC spectra after 120 min incubation of compound **KA-232** with human liver microsomes in TRIS buffer pH=7.4 at 37ºC. **73.81%** of parent compound remained in the reaction mixture. Three metabolites **M1**, **M2** and **M3** were identified as well as the products (m/z= 382.18, m/z=445.23) of compound degradation in physiological buffers (see also Figure S1)

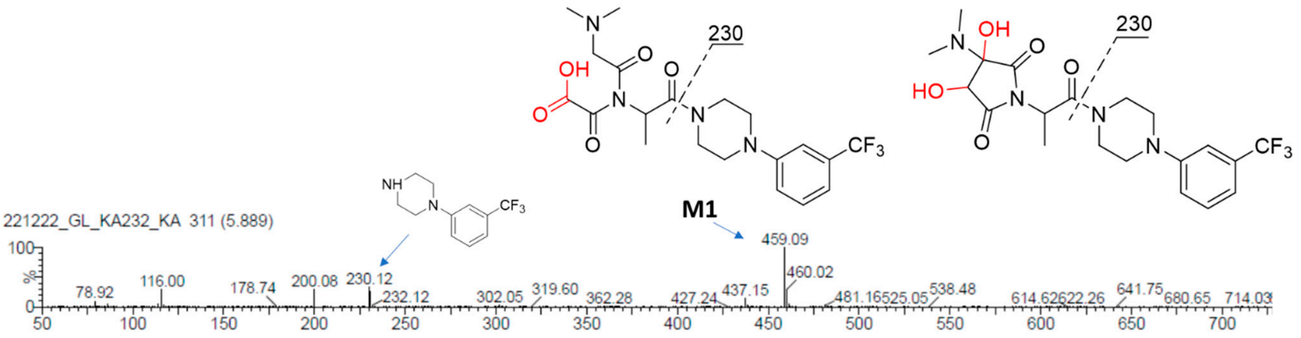

Figure S5 MS analysis and the proposed structures of **KA-232** metabolite **M1**.

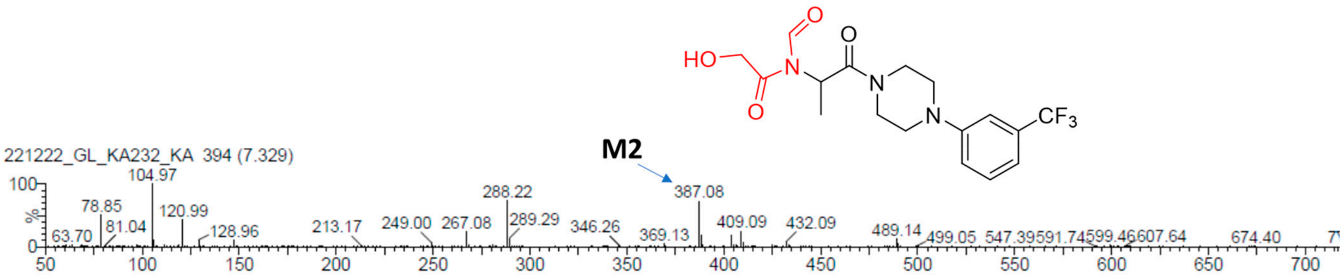

Figure S6 MS analysis and the proposed structure of **KA-232** metabolite **M2**.

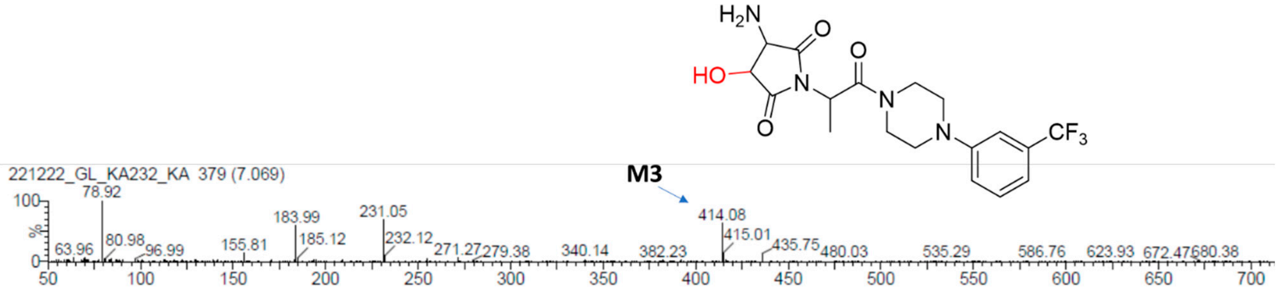

Figure S7 MS analysis and the proposed structure of **KA-232** metabolite **M3**.
